# Science or Fiction: Use of Nesiritide as a First-Line Agent?

**DOI:** 10.1592/phco.23.8.1081.32882

**Published:** 2012-01-16

**Authors:** John A Noviasky, Michael Kelberman, Karen M Whalen, Roy Guharoy, William Darko

**Affiliations:** Department of Pharmacy, St. Elizabeth Medical CenterUtica, New York; Cardiac Catheterization Laboratory, Mohawk Valley Heart InstituteUtica, New York; Department of Pharmacy, St. Joseph's HospitalSyracuse, New York; Department of Pharmacy, State University of New York Upstate Medical UniversitySyracuse, New York

Nesiritide (Natrecor; Scios Inc., Sunnyvale, CA), a recombinant form of human B-type natriuretic peptide, was recently approved by the Food and Drug Administration (FDA) for the treatment of acute decompensated chronic heart failure (CHF). This novel agent is a valuable addition, as there have been no new therapies developed for this indication in more than a decade. Though originally rejected by the FDA,^1^ completion of the Vasodilation in the Management of Acute CHF (VMAC) trial^2^ has resulted in its approval and general use. Indeed, numerous reviews of this agent indicate that it is appropriate as first-line therapy for symptomatic patients with decompensated CHF.^1^, ^3^–^6^ We, however, dispute this place in therapy, using data from the VMAC trial^2^ and additional unpublished data provided to the FDA about the VMAC trial. Although other studies of nesiritide exist, the VMAC trial is the only one in which both randomized and blinded patients receiving nesiritide were compared with patients receiving active control (nitroglycerin).

## Safety of Nesiritide

The VMAC trial showed that patients receiving nesiritide had fewer symptomatic adverse effects than those receiving nitroglycerin. The main difference was more frequent headache associated with nitroglycerin (20%) compared with nesiritide (8%; p<0.001). In addition, there was a small difference favoring nesiritide for less abdominal pain. Although headache is an adverse effect that should be of concern, it is not clear to what degree it affected patient care, as the number of patients who discontinued therapy due to headache was not included in the study results. On the other hand, a 50% relative-risk increase in patient dropout due to adverse effects was reported for patients receiving nesiritide (4.8%) compared with nitroglycerin (3.2%).^2^

## Efficacy of Nesiritide

**Figure 1:**
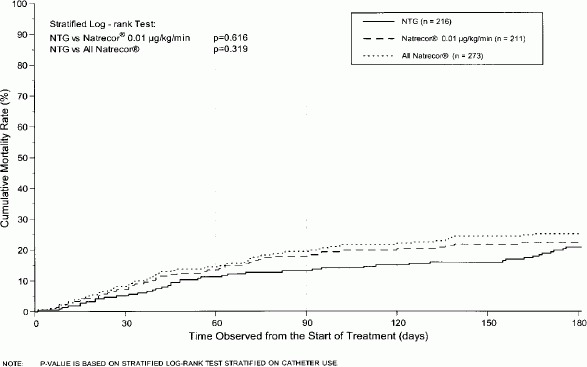
Kaplan-Meier estimate of mortality rate by treatment group (all treated subjects, as randomized).

Figure 1 depicts a Kaplan-Meier curve that clearly shows a trend toward decreased survival with the use of nesiritide.^7^ Indeed, the mortality assessment at 90 days favored nitroglycerin, with 27 deaths (13%), compared with 52 deaths (19%) associated with nesiritide (p=0.08). The authors of the VMAC trial felt that this difference was the result of an imbalance between the study groups; more patients in the nesiritide group were also receiving long-term therapy with class III antiarrhythmic agents. They also received an intravenous vasoactive drug within 24 hours of receiving the study drug and had the study drug added to their ongoing therapy with dobutamine or dopamine. Although these factors could be a consideration, results of a Cox regression analysis containing these variables did not appear to change the hazard ratio greatly.^8^

In patients who were receiving active drug and had right-heart catheters (180 patients [37%]), both nesiritide and nitroglycerin significantly decreased pulmonary capillary wedge pressure (PCWP) 5.9 ± 0.5 mm Hg (p≤0.001) and 3.9 ± 0.7 mm Hg (p=0.045), respectively, compared with placebo.^8^ Whereas it appears that the decrease in PCWP with nesiritide was larger than that with nitroglycerin, it should be noted that the nitroglycerin dosage was determined by investigators to achieve desired clinical effects.^8^ Thus any shortcomings in efficacy variables should take this into account. Moreover, there appears to be a dose-response relationship with nitroglycerin and PCWP. This has been shown with nitroglycerin infusions of 31–60 μg/minute, which produced a decrease in PCWP of 3 ± 3 mm Hg at 1 hour and 2 ± 3 mm Hg at 3 hours. When nitroglycerin was infused at a higher concentration (> 60 μg/min), the PCWP decreased by 5 ± 4 mm Hg at 1 hour and 7 ± 5 mm Hg at 3 hours.^9^ Indeed, this effect appears to be consistent with that shown with nesiritide at similar time periods, with decreases of 5.5 ± 6.3 and 5.8 ± 6.5 mm Hg at 1 and 3 hours, respectively.^2^

Regarding other clinical variables, the difference in the 3-hour dyspnea rating was not statistically significant between groups (p=0.6); in addition, no significant difference in dyspnea was found at 6 hours (p=0.37) and 24 hours (p=0.13).^8^ Although the difference in global clinical status was not statistically significant between patients receiving nesiritide and nitroglycerin at 3 hours (p=0.33) and 6 hours (p=0.12), a statistically significant difference was demonstrated at 24 hours (p=0.075). However, subgroup analysis showed this difference in global clinical status to be absent in catheterized patients (p=0.99).^8^

## Economic Considerations

The economic impact of a drug must take into consideration both the purchase price of the agent and its impact on patient outcome. Whereas there is no question that the cost of nesiritide is much higher (∼40 times) than that of nitroglycerin, improvements in morbidity and mortality rates could potentially offset the investment. Unfortunately, this does not appear to be the case. Although nesiritide generally requires less dosage titration than nitroglycerin, this factor alone cannot correct for the original expenditure.

In addition to these economic factors, close attention should be paid to the 2-day increased length of stay found with nesiritide compared with nitroglycerin in the VMAC trial.^7^ This length-of-stay increase coupled with using a more expensive drug regimen hardly seems to make economic (or common) sense. Although patients in the nesiritide group may have been more impaired in the VMAC trial, as mentioned earlier, this finding still needs to be addressed.

Another area that has a great impact on resource use is hospital readmission. In the VMAC trial, there was a trend toward decreased 30-day readmissions in patients receiving nesiritide (20%) versus those receiving nitroglycerin (23%).^2^ This trend was also seen with readmissions for acute decompensated CHF, which occurred in 13% of the nitroglycerin group and 7% of the nesiritide group. Although both of these differences seem to favor patients receiving nesiritide, a possible reason for the differences could be that mortality and length of stay were both increased in the nesiritide group. In other words, more nesiritide patients died and were in the hospital longer, thus becoming less likely candidates for hospital readmission.

## Summary

Nesiritide is an effective agent for the treatment of decompensated CHF. However, the VMAC trial shows that the agent's efficacy and safety are actually more similar than dissimilar to those of nitroglycerin. Indeed, objective reviews^10^–^11^ have placed nesiritide as a second-line agent behind current standard drug therapy. Finally, nesiritide is approximately 40 times the purchase price of standard agents such as nitroglycerin. For these reasons, we feel that nesiritide should not be considered as first-line therapy. Reflecting this notion, one institution has implemented a protocol that recommends administration of nitroglycerin and intravenous diuretics (using ≥2 times the usual daily diuretic dose) before using nesiritide.^12^ In light of the existing data, we feel that this approach appears to be an appropriate and prudent one for nesiritide's place in therapy.
